# Toxicity and Sublethal Effect of Farnesyl Acetate on Diamondback Moth, *Plutella xylostella* (L.) (Lepidoptera: Plutellidae)

**DOI:** 10.3390/insects12020109

**Published:** 2021-01-27

**Authors:** Norazila Yusoff, Idris Abd Ghani, Nurul Wahida Othman, Wan Mohd Aizat, Maizom Hassan

**Affiliations:** 1 Institute of Systems Biology, Universiti Kebangsaan Malaysia, Bangi 43600 UKM, Selangor, Malaysia; azilayusoff86@gmail.com (N.Y.); wma@ukm.edu.my (W.M.A.); 2 Centre for Insect Systematics, Department of Biological Sciences and Biotechnology, Faculty of Science and Technology, Universiti Kebangsaan Malaysia, Bangi 43600 UKM, Selangor, Malaysia; idrisyatie@yahoo.com.my (I.A.G.); wahida@ukm.edu.my (N.W.O.)

**Keywords:** *Plutella xylostella*, farnesyl derivatives, sublethal concentration, biological parameters, pest management, morphological abnormalities

## Abstract

**Simple Summary:**

The diamondback moth, *Plutella xylostella*, is the most destructive pest for *Brassica* vegetable crops worldwide. The management of this pest was estimated to cost about United States dollars (USD) 4–5 billion annually. Prolonged and unsupervised insecticide exposures have led to not only the emergence of insecticide resistance in *P. xylostella*, but also negative impacts on human health, environmental pollution, and nontargeted organisms. Therefore, the development of new safer, environmentally friendly, and target-specific insecticides is vital in order to combat this pest. In this study, we evaluated the potential of selected farnesyl derivative compounds that could act as biorational insecticides targeting the juvenile hormone biosynthesis of *P. xylostella.* Out of five farnesyl derivatives tested, farnesyl acetate showed the highest mortality percentage of *P. xylostella*. Then, the sublethal effects of farnesyl acetate on biological characteristics of *P. xylostella* were determined. The results demonstrated that farnesyl acetate had negative effects on development, pupal weight, pupation, adult emergence, female ratio, fecundity, egg hatching rate, and oviposition period of *P. xylostella*. Farnesyl acetate also induced abnormalities in pupal and adults of *P. xylostella*. These findings indicate that farnesyl acetate can reduce the population number and reproductive success of *P. xylostella*, possibly leading to the effective management of this pest.

**Abstract:**

The diamondback moth, *Plutella xylostella* (L.) (Lepidoptera: Plutellidae), is the most important pest of cruciferous vegetables worldwide. In this study, we evaluated the properties of selected farnesyl derivative compounds against *P. xylostella*. The toxicity and sublethal concentration (LC_50_) of farnesyl acetate, farnesyl acetone, farnesyl bromide, farnesyl chloride, and hexahydrofarnesyl acetone were investigated for 96 h. The leaf-dip bioassays showed that farnesyl acetate had a high level of toxicity against *P. xylostella* compared to other tested farnesyl derivatives. The LC_50_ value was 56.41 mg/L on the second-instar larvae of *P. xylostella*. Then, the sublethal effects of farnesyl acetate on biological parameters of *P. xylostella* were assessed. Compared to the control group, the sublethal concentration of farnesyl acetate decreased pupation and emergence rates, pupal weight, fecundity, egg hatching rate, female ratio, and oviposition period. Furthermore, the developmental time of *P. xylostella* was extended after being exposed to farnesyl acetate. Moreover, the application of farnesyl acetate on *P. xylostella* induced morphogenetic abnormalities in larval–pupal intermediates, adults that emerged with twisted wings, or complete adults that could not emerge from the cocoon. These results suggested that farnesyl acetate was highly effective against *P. xylostella*. The sublethal concentration of farnesyl acetate could reduce the population of *P. xylostella* by increasing abnormal pupal and adults, and by delaying its development period.

## 1. Introduction

The diamondback moth, *Plutella xylostella*, is a damaging pest for *Brassica* vegetable crops all over the world [1,2]. The management of this pest was estimated to cost about United States dollars (USD) 4–5 billion annually [3,4]. Relying on temperature, humidity, and food availability, the life cycle of *P. xylostella* takes between 18 and 51 days [5]. Due to the voracious appetite of the larvae, *P. xylostella* infestation can eradicate the entire cropping systems and render regions inadequate for production [6]. Rapid insect life cycle, high fecundity, high reproduction rate, and high selection pressure with insecticides are the major factors that contribute to the resistance of this pest to various insecticides [6,7]. Furthermore, its mobility and rapid resistance evolution against many insecticides become the ultimate drawbacks in managing this pest [8]. To boost crop production, farmers usually conduct more frequent sprays of insecticide, increase the doses, or apply mixtures of several insecticides. Prolonged and unsupervised insecticide exposures have led to not only the emergence of insecticide resistance in *P. xylostella*, but also negative impacts on human health, environmental pollution, and nontargeted organisms [9].

Juvenile hormones (JHs) are critical insect hormones that regulate a large diversity of physiological processes and adult reproduction in insects. Due to these roles in insects, JHs have gained attention as safe and specific targets for environmentally friendly and biorational insecticide discovery [10]. The juvenile hormone analogues (JHA), which are insect growth regulators (IGRs) that affect insect development and reproduction, have been developed and used in integrated pest management (IPM). JHAs are potent inhibitors of embryogenesis, metamorphosis, and adult formation of most insects. JHAs insecticides such as methoprene, fenoxycarb, and pyriproxyfen are commonly found in flea treatments for dogs and cats, mosquito control products, and insecticides against Lepidopteran pests [11,12,13] The application of JHAs such as fenoxycarb and pyriproxyfen have shown effectiveness on *P. xylostella* by suppressing adult emergence, fecundity, egg hatching, and abnormality in the pupal and adult insects [14,15,16]. However, previous studies reported that these insecticides also affected the development, behavior, and reproduction in beneficial insect and nontarget organisms [17,18,19,20]. Thus, a different strategy has to be envisaged for the development of new, safer, and efficient JH-based control products targeting *P. xylostella.* For this reason, interfering with JH biosynthesis has been considered as a promising strategy for the insecticide discovery [21]. The early steps in the biosynthetic pathway of juvenile hormone III (JHIII) follow the mevalonate pathway to form farnesyl pyrophosphate from acetyl-CoA [22]. The later steps involve the conversion of farnesyl pyrophosphate to farnesol through hydrolysis. Subsequently, farnesol is converted to farnesal and farnesoic acid via oxidation reactions. Farnesoic acid then undergoes an epoxidation and methyl transfer to form a JHIII [22]. Recent studies reported the characterization and inhibition of several enzymes from the JH biosynthesis pathway in insects, such as *Aedes aegypti* (L.), *Diploptera punctata* (Eschscoltz)*, Helicoverpa armigera* (Hubner), and *Manduca sexta* (L.). Characterization of recombinant juvenile hormone acid methyl transferase (JHAMT), which is an enzyme that converts juvenile hormone acid into JHIII, has shown that knockdown of JHAMT in *D. punctata* results in decreased oocyte and vitellin content as a consequence of reduction in JH biosynthesis [23]. In *H. armigera*, the knockdown of farnesyl diphosphate synthase caused the reduction of JH titer and, thus, disrupted the molting process in larvae [24]. Meanwhile, the characterization of enzymatic activity of JHAMT in *A. aegypti* revealed that several juvenile hormone acid analogues were potent inhibitors of JHAMT [25]. An enzyme-inhibitory activity of farnesol dehydrogenase in *M. sexta* was also observed, and geranylgeraniol caused a reduction in JH biosynthesis in vitro and mortality of larvae in a feeding toxicity test [26]. Previously, we characterized the farnesol dehydrogenase from *P. xylostella* and several farnesyl derivatives shown as potent analogue inhibitors for *P. xylostella* farnesol dehydrogenase when tested in vitro [27]. Farnesol dehydrogenase, an intermediate enzyme in the JHIII biosynthesis pathway, catalyzes the oxidation of farnesol to farnesal [28]. Farnesol dehydrogenase activity was observed in several insects [27,29] and plants [28,30,31]. A few studies reported that geranyl and farnesyl derivatives showed inhibitory effects on several insect pests [32,33,34,35]. Therefore, the objective of this study was to evaluate the toxicity of selected farnesyl derivatives against *P. xylostella* and the effects of sublethal concentration on developmental time, oviposition period, pupal weight, pupation, adult emergence, female ratio, fecundity, and egg hatchability. The findings from this study are expected to help develop new effective and safe insecticides to manage *P. xylostella* populations in particular and Lepidopteran pests in general.

## 2. Materials and Methods 

### 2.1. Insect Rearing

The larvae of *P. xylostella* were collected from vegetable farms in Cameron Highlands, Pahang, Malaysia (4°34′55″ N, 101°24′41″ E) and brought to the laboratory of Institute of Systems Biology, Universiti Kebangsaan Malaysia for rearing. Larvae were reared on mustard plant, *Brassica rapa*, as food in a screen cage (35 cm × 35 cm × 47 cm) at 25 ± 1 °C with a photoperiod of 12 h/12 h (light (L)/darkness (D)). The newly emerged adults were transferred to new mustard plants for oviposition, and cotton wool soaked in 10% honey water placed inside the cage was added as a dietary supplement [36]. The experiments were started after the third generations of the moth.

### 2.2. Bioassay and Determination of Sublethal Concentration (LC_50_) of Farnesyl Derivatives

The leaf-dip method was used for bioassay experiments [14] using five farnesyl derivatives: farnesyl acetate, farnesyl acetone, farnesyl bromide, farnesyl chloride, and hexahydrofarnesyl acetone. These farnesyl derivatives were selected because they bear a similar structure to farnesol and acted as potent analogue inhibitors for *P. xylostella* farnesol dehydrogenase when tested in vitro [27]. Four concentrations (12.5, 25, 50, and 100 mg/L) of each derivative were prepared by mixing with 0.02% Tween-20 and distilled water. Mustard leaf discs (4.5 cm diameter) were dipped in each concentration of farnesyl derivative solutions for 10 s. For the control treatment, the leaf discs were dipped in distilled water mixed with 0.02% Tween-20. The treated leaf discs were air-dried at room temperature for 2 h and then placed in a petri dish. A total of 10 second-instar larvae were released on the leaf discs inside the dish. The treatments (farnesyl derivatives and control) were replicated three times and arranged following a complete randomized design (CRD). The mortality of the larvae was observed and recorded after 96 h exposure to the treatments. The insects were considered dead when they were unable to move after being prodded with a soft paint brush [37].

### 2.3. Sublethal Treatment and Effects on P. xylostella

A farnesyl acetate solution at an LC_50_ concentration of 96 h (56.41 mg/L) (determined as above) was prepared and sprayed onto fresh mustard plants. After being air-dried for about 2 h, 100 second-instar larvae of *P. xylostella*, which were starved for about 2 h earlier, were released on farnesyl acetate-treated plants as mentioned above. For the control treatment, the mustard plants were sprayed with distilled water. Each treatment (LC_50_ of farnesyl acetate and control) was replicated three times, and the experiment was arranged as a CRD. The surviving larvae were transferred to new untreated mustard plants after 96 h exposure to the treated plants and allowed to continue their development until the pupal stage. The number of pupae was recorded daily until all larvae pupated. Pupae were placed individually in Petri dishes, and the 2 day old age pupae were weighed individually. The number of emerged pupae were recorded daily until all pupae emerged as adults. After adult emergence, 10 pairs of adults of each treatment were selected, and each pair (male and female emerged on the same day) was put in a new cage. For oviposition, adults were allowed to mate and lay eggs on mustard leaves placed in each cage. The number of eggs laid by each female adult was recorded daily, and the leaves were replaced every day until the death of the adults. Egg hatching was also recorded daily. The pre-oviposition period (time from adult emergence and first egg laying), oviposition period (time from first egg laying to last egg laying), and post-oviposition period (time from last egg laying to death) were also recorded. The pupation, emergence, female ratio, fecundity (total number of eggs laid per female), and egg hatching rate were calculated as described in Table 1.

### 2.4. Data Analysis

Percentage larval mortality was corrected following Abbott’s formula [42]. Data were transformed using arcsin to get normality before being analyzed with two-way analysis of variance (ANOVA). Probit analysis was performed using EPA Probit Analysis Program (version 1.5) to estimate the sublethal concentration (LC_50_) of farnesyl derivatives at 96 h. A *t*-test was carried out to analyze the effect of farnesyl acetate on the development, pupation, emergence, female ratio, oviposition period, fecundity, and hatching rate of *P. xylostella.* Significance was accepted at *p* < 0.05.

## 3. Results

### 3.1. Sublethal Concentration of Selected Farnesyl Derivatives on P. xylostella

**Figure 1:**
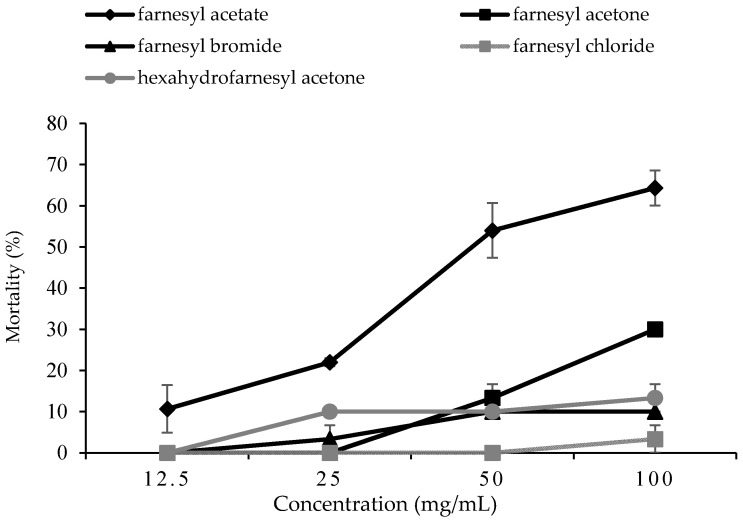
Percentage mortality of *P. xylostella* treated with farnesyl derivatives at various concentrations at 96 h. Data are shown as the mean ± standard error (SE).

The results of the bioassay showed that treatment of farnesyl derivatives significantly caused various levels of larval mortality (F = 118.309, degrees of freedom (df) = 4, *p* < 0.0001) (Figure 1). The larval mortality of each treatment was corrected to control mortality (Supplementary Data S1). The highest larval mortality (64%) was observed when *P. xylostella* were treated with 100 mg/L of farnesyl acetate, followed by farnesyl acetone (30%), hexahydrofarnesyl acetone (13.3%), farnesyl bromide (10%), and farnesyl chloride (3.3%). The larval mortality also significantly increased with increasing concentrations of farnesyl derivatives (F = 70.202, df = 3, *p* < 0.0001). A significant interaction (F = 14.73, df = 12, *p* < 0.0001) occurred between farnesyl derivatives and concentration in influencing the larval mortality. The estimated LC_50_ and LC_90_ at 96 h of farnesyl acetate were 56.41 and 272.56 mg/L, respectively (Table 2). Meanwhile, the LC_50_ and LC_90_ of farnesyl acetone were 142.87 and 407.67 mg/L, respectively. However, other farnesyl derivatives (farnesyl bromide, farnesyl chloride and hexahydrofarnesyl acetone) showed no toxicity effects on *P. xylostella* larvae.

### 3.2. Sublethal Effects of Farnesyl Acetate

#### 3.2.1. Developmental Time and Pupal Weight

Larval developmental time was significantly extended from 8.4 days (control) to 11.85 days in farnesyl acetate treatment (Table 3). Meanwhile, the development time of pupae was also significantly extended by the sublethal dose of farnesyl acetate compared to the control (*p* < 0.0001, *t* = −4.43, df = 58). However, the adulthood period was slightly shorter than that in the control treatment (*p* = 0.001, *t* = 3.55, df = 58). Treatment of farnesyl acetate caused a decrease in pupal weight of *P. xylostella* (Table 4). The pupal weight was significantly reduced by 1.41-fold compared to the control group.

#### 3.2.2. Pupation and Adult Emergence

The percentage pupation in *P. xylostella* treated with farnesyl acetate was significantly lower than that in the control group. The pupation rate was only 66.67%, 1.35-fold lower than the control group (Table 4). The adult emergence rate also reduced with farnesyl acetate treatment, which was 1.57-fold lower than the control (*p* = 0.03, *t* = 3.27, df = 4) (Table 4).

#### 3.2.3. Female Ratio, Fecundity, and Hatchability

A sublethal concentration of farnesyl acetate significantly reduced the *P. xylostella* female ratio (*p* = 0.007, *t* = 5.11, df = 4), fecundity (*p* < 0.0001, *t* = 17.68, df = 16), and egg hatching rate (*p* = 0.002, *t* = 3.82, df = 16) (Table 4). The female ratio decreased from 45.3% in the control to 27.47% in the treated group. Meanwhile, the fecundity and egg hatching rate reduced by 4.17- and 1.10-fold, respectively.

#### 3.2.4. Ovipositional Period

Farnesyl acetate at LC_50_ significantly delayed the pre-oviposition period (*p* = 0.018, *t* = −2.64, df = 16), which was 0.45 days longer than the control group (Table 5). Meanwhile, both the oviposition and the post-oviposition periods were significantly shorter compared with those of the control group. The oviposition and post oviposition durations were 3.11 days and 0.75 days shorter than the control group.

#### 3.2.5. Abnormalities Caused by Farnesyl Acetate Treatment

**Figure 2:**
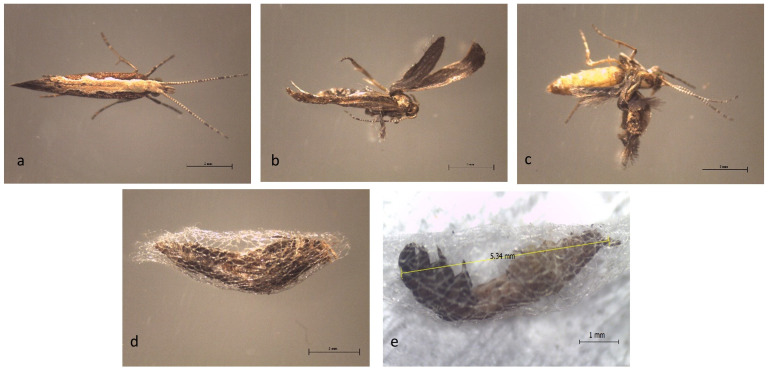
Morphological abnormalities in treated *P. xylostella* with sublethal concentration of farnesyl acetate at adult and pupal stages: (**a**) normal adult; (**b**,**c**) adult with twisted wings; (**d**) adult trapped in the cocoon; (**e**) larvae–pupae intermediates; scale bar: 1 mm.

Farnesyl acetate also induced malformation and abnormalities of *P. xylostella* morphology, including the formation of larval–pupal intermediates, complete adults that could not emerge from the cocoon, and adults with twisted wings (Figure 2).

## 4. Discussion

This study evaluated the sublethal concentration of selected farnesyl derivatives against *P. xylostella*. The leaf-dip bioassay revealed that farnesyl acetate showed the highest toxicity and lowest LC_50_ against *P. xylostella* compared to other tested farnesyl derivatives. The results also indicated that the LC_50_ of farnesyl acetate was 4 to 100-fold higher than that of several broad-spectrum insecticides and IGRs such as spinosad, fenvalerate, fipronil, methomyl, teflubenzuron and hexaflumuron [40,43,44,45,46,47] (Table 6). Unfortunately, *P. xylostella* has developed resistance to most of those insecticides. On the other hand, the LC_50_ of farnesyl acetate was 2 to 700-fold lower than methamidophos, diflubenzuron and dichlorodiphenyltrichloroethane (DDT) [46,48]. Similarly, the LC_50_ of farnesyl acetate was 1.66- and 35.63-fold lower than commercially available JHAs, fenoxycarb and pyriproxyfen, respectively [15,16]. The use of a lower concentration of insecticides with high sublethal effects constitutes an environmentally friendly alternative for improving IPM strategies [49]. Furthermore, a recent finding reported that farnesyl acetate exhibited larvicidal activity and induced inhibition of ovary growth in mosquito, *Aedes albopictus* (Skuse), by modulating the formation and expression of the JH receptor complex [35]. The LC_50_ concentration of farnesyl acetate was then selected for the study of the sublethal effects on *P. xylostella.*

The treatment with the LC_50_ of farnesyl acetate on *P. xylostella* affected the developmental period and process of *P. xylostella*. The larval and pupal periods of *P. xylostella* were extended after the application of farnesyl acetate. Previous studies reported that the treatment of JHAs, pyriproxyfen, and fenoxycarb extended the developmental period of larval and pupal stages of the *P. xylostella* [14,15,16]. On the other hand, the application of a sublethal concentration of hexaflumuron, an IGR, on *P. xylostella* larvae also prolonged larval and pupal developmental time [49]. Other studies of JHA effects on insects such as beet armyworm (*Spodoptera exigua* (Hubner))*,* obliquebanded leafroller (*Choristoneura rosaceana* (Harris)), Indian meal moth (*Plodia Interpunctella* (Hubner)), Asian citrus psyllid (*Diaphorina citri* (Kuwayama)), and lesser grain borer (*Rhyzopertha dominica* (Fabricius)) also revealed that JHAs extended the development time of the insects [50,51,52,53,54]. The extension of the larval or pupal period might be because of the prolonged presence of JH in the hemolymph [55]. Therefore, ecdysone may not be triggered, interrupting the formation of the next stage. The application of pyriproxyfen on *Tenebrio molitor* (L.) significantly inhibited the production of ecdysone and, therefore, interfered with the normal development of this insect [56]. Despite the extension of larval duration, the pupal weight decreased following the treatment of farnesyl acetate. The treatment of pyriproxyfen on *P. xylostella* also reduced pupal weight due to impaired amino-acid uptake, lipid synthesis, and catabolism [15]. Similarly, fenoxycarb severely interrupted lipid synthesis and catabolism, and it decreased food consumption and development rate in silkworms, *Bombyx mori* (L.) [57]. Hence, the reduction in pupal weight of *P. xylostella* in this study can perhaps be attributed to disturbed nutrition intake, lipid synthesis and catabolism.

A sublethal concentration of farnesyl acetate had a negative effect on pupation and adult emergence rate of *P. xylostella*. Treatment of JHA also showed a decreasing adult emergence rate of *P. xylostella*. The emergence rate in *P. xylostella* was reduced from 96% in the control group to 76% as a consequence of pyriproxyfen treatment [15]. In Mediterranean flour moth (*Ephistea kuehniella* (Zeller)), the pupation and emergence rates were very low when fenoxycarb was applied to the last instar of the insect larvae [58]. Treatment of cotton whitefly, *Bemisia tabaci* (Gennadius) with pyriproxyfen demonstrated a similar pupation rate to the control, but adult emergence was reported to be less than 10% [59]. Meanwhile, only 0–36% of early-instar and 25–74% of late-instar Asian citrus psyllid, *D. citri*, survived to adults after exposure to pyriproxyfen [53]. Adult emergence of stored grain pests, *Oryzaephilus surinamensis* (L.)*, Tribolium castaneum* (Herbst), and *Trogoderma granarium* (Everts), was also reduced when exposed to pyriproxyfen [60].

Previous studies on *P. xylostella* and *S. exigua* demonstrated that there were no differences in female ratio after the treatment of a JHA, pyriproxyfen [15,51]. However, the application of JHAs has been known to influence the reproductive ability of insects. The fecundity and egg hatchability of *P. xylostella* decreased after being treated with pyriproxyfen and fenoxycarb [14,15,16]. Similar effects were observed in *C. rosaceana*, *S. exigua*, *D. citri* and *Platynota idaeusalis* (Walker) [50,51,53,61] as a result of treatment with JHAs. The egg hatching suppression was also observed in *T. castaneum* and *T. granarium* due to pyriproxyfen treatment [62]. In this study, *P. xylostella* reproduction may have been negatively affected by smaller (underweight) pupae that failed to consume enough nutrients and underwent abnormal physiological processes for growth. The nutritional condition of the female can influence the development of ovaries and egg production [63]. The reduction in macronutrient compounds such as lipids, proteins, and carbohydrates consumed by the insects may result in abnormal oogenesis [64]. The incorporation of pyriproxyfen was reported to affect the ovary’s development and oogenesis, reducing the fecundity and egg hatching rate of *P. interpunctella* [52]. Another JHA, methoprene, also affected the ovarian growth of *Blatta germanica* L., causing a drastic decrease in oocytes in newly emerged adults [65].

The ovipositional period plays an important role in the reproductive success of insects. A decreased oviposition time may result in a decrease in fecundity and vice versa. In the present study, the pre-oviposition period of *P. xylostella* was extended but the oviposition period was shortened compared to the control. The results are similar to the previous study of *P. xylostella*, indicating that a JHA, pyriproxyfen, delayed the pre-oviposition period of *P. xylostella* but shortened the oviposition period [14]. The treatment of pyriproxyfen slowed the maturation of the ovaries, thereby postponing adult mating [14]. In contrast, another study reported that pyriproxyfen prolonged the oviposition period of *P. xylostella* compared to the control [15]. The treatment of *P. xylostella* with other insecticides such as hexaflumuron (IGR), lufenuron (IGR), and cantharidin (natural toxin from blister beetle) also reduced the oviposition period [46,66,67].

The abnormalities of *P. xylostella* morphology observed in this study are in accordance with the previous study on a JHA, pyriproxyfen. The treatment of *P. xylostella* larvae with a sublethal dose of pyriproxyfen triggered abnormalities in *P. xylostella*, such as untanned pupae, larvae–pupae intermediates, imperfectly sclerotized pupae, adults that were unable to emerge, and adults with twisted winged [15]. Similar morphogenetic abnormalities were reported in *S. exigua* and *D. citri* as a consequence of pyriproxyfen treatment [51,53]. At the pupal stage, only ecdysone is released by the insects and JH is absent to allow metamorphosis to occur [68]. Hence, the insect is unable to excrete the excess JH through the introduction of external JHA during the larval period. Consequently, the presence of JHA may induce inhibition of ecdysone production, thereby disturbing the normal larval–pupal molt commitment, and leading to larval–pupal or larval–adult intermediates and other morphological abnormalities [50,56].

## 5. Conclusions

Our results suggest that farnesyl acetate is highly effective against *P. xylostella*. A sublethal concentration of farnesyl acetate had negative effects on *P. xylostella*, such as development, pupal weight, pupation, emergence, fecundity, egg hatchability, and oviposition period. Farnesyl acetate also caused abnormalities in pupae and adults of *P. xylostella*. This led to low reproductive success and reduced the population number and possibly the infestation level, resulting in good IPM of *P. xylostella*. However, further works are required to assess the sublethal effects of farnesyl acetate on several generations, and intensive studies are needed to determine the sublethal effect on field populations of insects.

## 6. Patent

Farnesyl acetate has been filed for a patent (Malaysia Patent Office (MyIPO), Application No. PI2018002697 entitled “An Insect Composition”.

## Figures and Tables

**Table 1:** Calculation of sublethal effects properties of *Plutella xylostella.*

Sublethal Effects	Calculation	References
(i) Pupation rate (%)	$\frac{Number\;of\;larvae\;survived\;until\;pupation}{Total\;number\;of\;larvae\;in\;the\;test}$ ×100	[38]
(ii) Emergence rate (%)	$\frac{Number\;of\;pupae\;survived\;until\;adult\;emergence}{Total\;number\;of\;larvae\;survived\;until\;pupation}$ ×100	[38]
(iii) Female ratio (%)	$\frac{Number\;of\;female\;adults}{Total\;number\;female\;+\;male\;adults}$ ×100	[39]
(iv) Fecundity	*Total number of eggs laid per female*	[40]
(v) Hatching rate (%)	$\frac{Number\;of\;hatching\;eggs}{Total\;number\;of\;eggs}$ ×100	[41]

**Table 2:** Toxicity of farnesyl derivatives on the second instar larvae of *P. xylostella.* LC_50_, sublethal concentration.

Farnesyl Derivatives	*N* ^1^	LC_50_	LC_90_	Slope ± SE	χ^2^	*p*-Value
Farnesyl acetate	150	56.41 (36.963–92.523)	272.562 (141.988–1696.881)	1.87 ± 0.487	0.943	>0.05
Farnesyl acetone	150	142.87 (94.116–849.911)	407.67 (186.36–23,669.26)	2.814 ± 0.991	1.019	>0.05
Farnesyl bromide	150	-	-	-		
Farnesyl chloride	150	-	-	-		
Hexahydrofarnesyl acetone	150	-	-	-		

^1^ Number of larvae tested.

**Table 3:** Effects of sublethal concentration of farnesyl acetate on the developmental period of *P. xylostella.* df, degrees of freedom.

	Developmental Time (Mean ± SE) (Days)		df	*t*	*p*
	Control	Treatment			
Second instar larvae	1.6 ± 0.08	2.6 ± 0.09	58	−8.06	<0.0001
Third instar larvae	1.6 ± 0.09	2.4 ± 0.09	58	−6.22	<0.0001
Fourth instar larvae	3.2 ± 0.07	4.85 ± 0.14	58	−10.65	<0.0001
All larvae	8.4 ± 0.17	11.85 ± 0.21	58	−12.00	<0.0001
Pupa	5.17 ± 0.17	6.17 ± 0.18	58	−4.43	<0.0001
Adult	21.4 ± 0.55	20.8 ± 0.45	58	3.55	0.001

**Table 4:** Effects of sublethal concentration of farnesyl acetate on pupal weight, pupation rate, adult emergence rate, female ratio, fecundity, and egg hatching rate (mean ± SE).

	Pupal Weight (mg)	Pupation (%)	Emergence (%)	Female Ratio (%)	Fecundity (Egg/Female)	Egg Hatching (%)
Control	7.4 ± 0.07	90 ± 1.15	92 ± 2.11	45.3 ± 1.17	104.78 ± 4.4	84.17 ± 1.12
Treated	5.23 ± 0.21	66.67 ± 7.69	58.7 ± 9.96	27.47 ± 3.3	25.11 ± 0.98	76.64 ± 1.62

**Table 5:** Effects of sublethal concentration of farnesyl acetate on pre-oviposition, oviposition, and post-oviposition periods (day ± SE) of *P. xylostella.*

	Mean ± SE (Days)		df	*t*	*p*
	Control	Treatment			
Pre-oviposition	0.9 ± 0.05	1.35 ± 0.16	16	−2.64	0.018
Oviposition	16.33 ± 0.44	13.22 ± 0.4	16	5.22	<0.0001
Post-oviposition	3.05 ± 0.19	2.3 ± 0.17	16	2.8	0.0123

**Table 6:** Sublethal concentration (LC_50_) of insecticides on *P. xylostella.* JHA, juvenile hormone analogue; IGR, insect growth regulator.

Class	Insecticides	LC_50_ (mg/L)	References
	Farnesyl acetate	56.41	In this study
	Farnesyl acetone	142.87	In this study
JHA	Pyriproxyfen	2010	[15]
	Fenoxycarb	93.92	[16]
IGR	Teflubenzuron	6.73–1440	[46]
	Diflubenzuron	1907–2171	[46]
	Hexaflumuron	1.48	[47]
Broad spectrum	Spinosad	0.28	[40]
	Fenvalerate	13-2700	[43]
	Fipronil	7.57	[44]
	Methomyl	0.4-7.3	[45]
	Methamidophos	116.35-6755	[46]
	DDT	170-44200	[48]

## Supplementary Materials

The following are available online at https://www.mdpi.com/2075-4450/12/2/109/s1, Data S1: Larval mortality.
